# Materia Medica and Therapeutics: With Ample Illustrations of Practice in All the Departments of Medical Science, and Very Copious Notices of Toxicology, Suited to the Wants of Medical Students and Practitioners

**Published:** 1850-11

**Authors:** 


*Materia Medica and Therapeutics: with Ample Illustrations of Practice in all the Departments of Medical Science, and very copious notices of Toxicology, suited to the wants of Medical Students and Practitioners.* By THOMAS D. MITCHELL, A. M., M. D., Prof. of the Theory and Practice of Medicine in the Philadelphia College of Medicine, etc., etc., etc. Philadelphia: Lippincott, Grambo & Co., successors to Grigg, Elliott & Co., 14 North Fourth-street. 1850. 8 vo., pp. 738.

This volume, as the author states in his *Introduction*, contains the substance of his Lectures on Materia Medica and Therapeutics in the Medical Department of Transylvania University for eleven successive winters. It differs from other treatises on the same subjects in being divested of dry details relating to the natural, botanical and chemical history of medicinal articles. In place of these the author gives the fruits of his own reflections and experience in the use of remedies, at the same time gleaning the observations of others from various sources, thus rendering the work eminently practical in its character. This feature will serve to commend it to the attention of the Practitioner. In so far as we may judge from a partial examination of its merits, it deserves a place in every medical library. Prof. M. adopts the alphabetical arrangement of the articles of the materia medica. For sale by Phinney & Co.

